# Genes and models for estimating genetic parameters for heat tolerance in dairy cattle

**DOI:** 10.3389/fgene.2023.1127175

**Published:** 2023-02-27

**Authors:** Vincent Habimana, Chinyere Charlotte Ekine-Dzivenu, Athumani Shabani Nguluma, Zabron Cuthibert Nziku, Gota Morota, Sebastian Wilson Chenyambuga, Raphael Mrode

**Affiliations:** ^1^ Department of Animal, Aquaculture and Range Sciences, Sokoine University of Agriculture, Morogoro, Tanzania; ^2^ SACIDS Foundation for One Health, College of Veterinary Medicine and Biomedical Sciences, Sokoine University of Agriculture, Morogoro, Tanzania; ^3^ International Livestock Research Institute (ILRI), Nairobi, Kenya; ^4^ Tanzania Livestock Research Institute (TALIRI), Eastern Zone, Tanga, Tanzania; ^5^ School of Animal Sciences, Virginia Polytechnic Institute and State University, Blacksburg, VA, United States

**Keywords:** genetic models, heat tolerance, milk production traits, physiological markers, temperature-humidity index

## Abstract

Dairy cattle are highly susceptible to heat stress. Heat stress causes a decline in milk yield, reduced dry matter intake, reduced fertility rates, and alteration of physiological traits (e.g., respiration rate, rectal temperature, heart rates, pulse rates, panting score, sweating rates, and drooling score) and other biomarkers (oxidative heat stress biomarkers and stress response genes). Considering the significant effect of global warming on dairy cattle farming, coupled with the aim to reduce income losses of dairy cattle farmers and improve production under hot environment, there is a need to develop heat tolerant dairy cattle that can grow, reproduce and produce milk reasonably under the changing global climate and increasing temperature. The identification of heat tolerant dairy cattle is an alternative strategy for breeding thermotolerant dairy cattle for changing climatic conditions. This review synthesizes information pertaining to quantitative genetic models that have been applied to estimate genetic parameters for heat tolerance and relationship between measures of heat tolerance and production and reproductive performance traits in dairy cattle. Moreover, the review identified the genes that have been shown to influence heat tolerance in dairy cattle and evaluated the possibility of using them in genomic selection programmes. Combining genomics information with environmental, physiological, and production parameters information is a crucial strategy to understand the mechanisms of heat tolerance while breeding heat tolerant dairy cattle adapted to future climatic conditions. Thus, selection for thermotolerant dairy cattle is feasible.

## 1 Introduction

Dairy cattle are adversely affected by the detrimental effects of extreme weather conditions. Heat stress occurs when extreme environmental parameters exceed dairy cattle thermal comfort zone (Hammami et al., 2013; Wang et al., 2018) and this significantly affects their heat dissipation rates, production and reproduction traits (Bohlouli et al., 2013). A lactating dairy cattle is affected by heat stress when she is unable to dissipate the extreme core body temperature and maintain unchanged internal body heat (Hill and Wall, 2014; Wang et al., 2018). Heat stress negatively affects livestock production, reproduction, health and general performance leading to substantial economic losses (West, 2003; Wang et al., 2018). Globally, heat stress impact on livestock production is beyond 1.2 billion dollars (Srikanth et al., 2017). High producing dairy cattle are sensitive to the effects of heat stress at the first stage of lactation and when a core body temperature exceeds 39°C, milk yield significantly decreases (Ravagnolo and Misztal, 2000). A heat stressed dairy cow drops milk production and feed intake by 17%–35%, and 35%–48%, respectively and has low conception rates in first two parities (Osei-Amponsah et al., 2020). Additionally, heat stress affects conception in dairy cows. Conception rates from artificial insemination for Australian Holstein Friesian dairy cattle have been found to be low, varying from 10% to ˂ 55% during the period of extreme climatic conditions (Osei-Amponsah et al., 2020). In addition to the above, changes in physiological parameters such as high core body temperature, rectal temperature, respiration rate, heart and pulse rates which are crucial for cow welfare and production performance have been associated with increased temperature (Garner et al., 2016). Response to changes in those physiological parameters are the cow’s coping mechanisms. For example, under hot conditions (temperature of 24–39°C and relative humidity of 32%–60%) an increase of 1°C to ambient temperature increases respiratory rates from 2.8 to 3.3 breaths per minute (Osei-Amponsah et al., 2020).

Owing to lack of weather station data on solar radiation and wind speed, many researches have embarked on air temperature and relative humidity to assess heat stress effects on dairy cattle performance (Pryce et al., 2022).

Temperature and humidity (often combined as a THI) is generally used to quantify the level of heat stress in animals. The temperature-humidity index combines the effects of dry temperature and relative humidity associated with the degree of heat stress. It has been developed as a climate index to determine and reduce losses associated to heat stress (Bohmanova et al., 2007). In dairy cattle, THI that exceeds a particular threshold for cattle, often leads to significant declines in feed intake, extreme water consumption, alteration in milk yield traits, and lowered fertility (Gálik et al., 2021; Wang et al., 2022).

Globally, approximately 60% of dairy farms are in heat-stress environments. The impacts of heat stress in dairy cattle can be addressed by providing shades, fans, and sprinklers to the animals. However, these management practices may not be practical in pastoral and agro-pastoral production systems which are the dominant systems in sub-Saharan Africa. Selection for heat tolerance, which have cumulative and permanent effects, could be a better strategy in these production systems. It has been shown that heat tolerance among dairy cattle breeds varies significantly (Osei-Amponsah et al., 2020). Identification and selection of heat-tolerant animals is an important strategy for minimizing the heat stress effects on dairy cattle productivity (Rong et al., 2019; Sigdel et al., 2019). Thus, it is crucial to include heat adaptive parameters in the selection objective of dairy cow population. Most genetic researches on climate change effects in dairy cattle have focused on 1) assessing the heat stress threshold for which milk yield begins to decline and 2) regressing phenotypic performance on THI value to quantify the genetic parameters of thermo-tolerance. The above modeling approaches assume that all cows have similar THI value and decrease rate in every environment and time (Hammami et al., 2015). It has been suggested that modeling cow performance as a model of a continuous THI can enable the identification and selection of high performing individuals, but with minimum sensitivity to THI (Hammami et al., 2013). Unfortunately, optimization of models that describe heat stress effects in dairy cows has not received much attention. Traditional models for describing an animal’s production performance in response to increased heat stress, known as broken line (BL) model, assumes that production doesn’t change in thermoneutral zone, and after the threshold point, the production decreases linearly (Carabaño et al., 2016). An alternative is to model the animal’s productive response using a reaction norm which uses polynomials. This approach offers higher flexibility than the BL (Pryce et al., 2022). On the other hand, various studies have indicated associations underlying genomic regions and heat tolerance traits in lactating cows (Sigdel et al., 2019). For instance, Srikanth et al. (2017) used RNAseq-analysis to detect genes involved in apoptosis, immune responses and metabolic pathways as candidate genes responsible for response to heat stress in Holstein dairy cattle. In another study, Cheruiyot et al. (2021) used genome wide association studies (GWAS) to identify candidate causal variants and genes responsible for thermotolerance in Australian dairy cows. Recently, Macciotta et al. (2017) and Sigdel et al. (2019) also detected candidate genes and genomic regions harboring heat tolerant genes associated with milk production traits under varying climatic conditions. Evidence from cow responses to heat stress in those studies indicate that genetic selection for heat tolerance in lactating dairy cattle is feasible (Ravagnolo and Misztal, 2000).

In this review, we discussed 1) heat tolerance and its importance, particularly in dairy industry, 2) indicators of heat stress, 3) genetic models for estimating genetic parameters for heat tolerance, 4) genes responsible for heat tolerance in dairy cattle, 5) genetic parameters for heat tolerance in dairy cattle and 6) genetic relationships between indicators of heat tolerance and performance traits.

## 2 Selection for heat tolerance and its importance

Heat tolerance is the ability to maintain thermal balance at extreme climatic conditions (Carabaño et al., 2019). Heat tolerance is a complex trait, and decline in production is only one potential indicator phenotype for this trait (Nguyen et al., 2016). Resilience to heat stress, is the ability of livestock species to be less sensitive to the climate change effects (Colditz and Hine, 2016) characterized by change in air temperature, relative humidity (Armstrong, 1994). This process is controlled by livestock ability to dissipate the heat produced by metabolic heat production (Pryce et al., 2022). In cattle, the heat stress value on which milk production starts to decline is 22 C and 69 units for average temperature and THI (Mbuthia et al., 2021).

Heat tolerance traits cause reduction in production decline. Thus, selection for thermotolerance has favourable effects on animal welfare (Polsky and von Keyserlingk, 2017). Heat tolerance is a heritable parameter (Ravagnolo and Misztal, 2000) in nature and genetic selection can be used to improve thermotolerance, as long as the phenotypes and tools are available for these selection decisions to happen. Heat tolerance traits for genetic selection could incorporate physiological traits, feed intake decline, decline in milk production traits and effects on reproductive traits related to THI (Pryce et al., 2022). Estimation of genetic parameters for milk production and reproductive traits in USA Holstein cattle have been found to rise at THI threshold of 72, which indicates that genetic selection for improving thermotolerance is feasible for milk production traits (Ravagnolo and Misztal, 2000).

Genetic component of heat tolerance is not negligible, thus selection for heat tolerance has to be incorporated in the selection objectives (Bernabucci et al., 2014). In an effort to improve heat tolerance in dairy cattle in Australia, Nguyen et al. (2016) developed genomic estimated breeding values (GEBV) for thermotolerance dairy cows through combining herd-test day records of Holstein and Jersey and daily THI data from public weather stations. This study indicated that selecting for heat tolerance increases milk production traits per unit of THI in Holsteins and Jersey dairy cattle but such increase varies by regions (Nguyen et al., 2016). Garner et al. (2016) used genome wide DNA markers to predict the responses of Holsteins dairy cattle to heat stress in Australia and found that milk production decline and other physiological traits was low compared to heat stressed dairy cattle. *Slick hair* gene initially identified in Senepol and Corora cattle was introduced in Holsteins through crossbreeding (Dikmen et al., 2008). Dikmen et al. (2014) indicated that rectal temperature, respiration rates and sweating rates were lower in slick cows than wild-type cows. Carmickle et al. (2022) reported that the presence of SLICK1 allele of the prolactin receptor gene was associated with low rectal temperature rates in pre and post-weaning Holstein females. Moreover, heat tolerance has a negative genetic correlation with milk production parameters (Aguilar et al., 2009). Thus, selection focusing on high producing dairy cattle is detrimental to heat tolerance (Pryce et al., 2022).

## 3 Indicators of heat stress

### 3.1 Temperature-humidity index

Temperature-humidity index is used as a biomarker of heat stress in dairy cow and incorporates the effects of air temperature and relative humidity (Bernabucci et al., 2014; Lambertz et al., 2014). The effect of THI on dairy cow performance is mainly affected by the breed type and the environment or location where the animal is reared. Also, the sensitivity of a cow to the effects of THI is affected by non-genetic factors, such as the lactation stage and the lactation number (Yan et al., 2021). Regarding breed-specific characteristics, Holstein dairy cattle have a higher increase in rectal temperature than low producing dairy cattle and the zebu breeds are heat tolerant to the effects of heat stress with regards to milk production and somatic cell count changes (Osei-Amponsah et al., 2020; Velayudhan et al., 2022). Furthermore, the variations in heat stress thresholds are based on the different environments, locations, or regions in which animals are reared as well as to the quantitative genetic models used in each study (Bernabucci et al., 2014; Mbuthia et al., 2021).

Livestock animals in various agro-ecological regions have different heat stress response values, and changes of genetic parameters above the thresholds have been identified (Velayudhan et al., 2022). In the study by Velayudhan et al. (2022), heat stress threshold for milk production traits identified is THI = 75, an indication that such value can be used in genetic evaluations for thermotolerance in dairy cows. In Thailand, the THI value of 76 was detected as heat stress threshold for dairy cattle (Sungkhapreecha et al., 2022). Thailand has a year-round THI ranging from 72 to 84 but in most cases THI is higher than 76 and this occurs for about 250 days in a year (Sungkhapreecha et al., 2022). This implies that dairy cattle reared in Thailand experience heat stress effects most of the year. In a study carried out in Bangladesh, average THI values observed in July to October ranged from 84.95 to 79.57, indicating an absence of cold stress, but with extreme heat stress effects for the study period starting from July to October (THI> 72). Extreme THI thresholds observed in those studies indicate that the majority of dairy cattle herds experience the adverse effects of climate change factors (Reyad et al., 2016). Another study performed on Holstein dairy cattle in three European countries, milk yield decline indicated an inverted U-shaped pattern in response to heat stress with THI threshold of 73 (Carabaño et al., 2016). A study conducted on smallholder farms in Tanzania, Ekine-Dzivenu et al. (2020) detected heat stress threshold value of 67.5. From the above findings from Asia, Europe and Africa, it is clear that THI threshold varies depending on the location and the breed genotypes. A THI value of 72 corresponding with 22°C and 100% relative humidity, has been continuously applied as a reference for genetic evaluation of heat tolerance in US Holsteins for daily milk (Hammami et al., 2013). THI can be used on a large-scale animal and at national level during genetic evaluation of heat tolerance in dairy cattle.

THI has been used for assessing losses of milk production in Holstein dairy cattle in the US (Bohmanova et al., 2007). They are classified based on breed genotypes, geographical location and genetic models used to calculate them. For instance, THI value = 71 is comfort zone, THI = 72 to 79 is low stress while THI = 80 to 89 is an average stress, and THI values greater than 90 is extreme stress in US Holstein Friesian dairy cattle (Armstrong, 1994). Other THI categories recently reported include 1) 68 ≤ THI <72 mild; 2) 72 ≤ THI <79 moderate; 3) 80 ≤ THI <89 severe; and 4) THI ≥90 is emergency heat stress for US Holstein Friesian dairy cattle reared in Arizona and California states (Wang et al., 2018).

THI is regarded as widespread marker of heat stress in animals, though it has its limitations because it is 1) an empirical representation, 2) assumes that all livestock species respond similarly to non-genetic stressors, and 3) does not account for other environmental parameters (e.g., wind speed and solar radiation although probably strictly correlated to THI) and animal specific factors (e.g., age and genotype) (Hammami et al., 2013). According to Bohmanova et al. (2007) there are seven formulae presented in Table 1 which are used to calculate THI with temperature in Celsius degrees while relative humidity is in percentage. Among the above formulae, THI_1_, THI_3_, and THI_6_ are used to monitor discomfort from temperature and relative humidity in humans while THI_2_ and THI_7_ are used to investigate heat stress effects for cattle exposed in climatic chambers. Only THI_5_ is used to determine the level of heat stress in dairy cattle reared outdoor (Bohmanova et al., 2007) with T_db_ and RH representing dry bulb temperature (^o^C), and relative humidity (%), respectively (Wang et al., 2022). Formulae used to calculate other environmental parameters (THI, adjusted temperature humidity index (THI), heat load index (HLI), equivalent temperature index (ETI), environment stress index (ESI), comprehensive climate index (CCI), relative humidity, wind speed, solar radiation and predicted globe temperature) are presented in Table 2, as reported in the study by Hammami et al. (2013). Public weather station data have an advantage in calculating THI based on their reliability, availability online and those stations do not rely on specific animal measurement of physiological parameters, which are difficult to generate at large scales (Lee et al., 2019). THI is easily calculated from the public station data and regressed with on-farm data. However, heat stress from solar radiation and wind speed data experienced by an animal are difficult to measure as they rely on specific tools and public weather stations do not record those parameters (Bohmanova et al., 2007). Moreover, weather station data is less effective in describing the conditions experienced by the cow in herds that have effective mitigation strategies, which suggests that more herd (cow specific) data will be necessary for effective estimation of heat tolerance from field data.

**TABLE 1:** Formulae used to calculate temperature-humidity index (THI).

No	Formula	References
1	THI_1_ = (0.15 x T*db* +0.85xT*wb*) x1.8 + 32	Bohmanova et al. (2007)
2	THI_2_ = (0.35x T*db*+0.65xT*wb*) x1.8 + 32	
3	THI_3_ = [0.4x (T*db* + T*wb*)]x1.8 + 32+15	
4	THI_4_ = (0.55 + T*db*+0.2 + T*dp*) x1.8 + 32+17.5	
5	THI_5_ = (1.8x T*db*+32)—(0.55–0.0055x RH) x (1.8xT*db*-26)	
6	THI_6_ = (T*db* + T*wb*) x0.72 + 40.6	
7	and THI_7_= (Tdb+0.36 x T*db*) +41.2	

^a^ T_db_ = dry bulb temperature (^o^C), RH, relative humidity (%).

**TABLE 2:** Formulae used to calculate environmental parameters.

No	Formula	References
1	THI = (1.8 × Tdb +32) − [(0.55–0.0055 × RH) × (1.8 × Tdb −26)]	Hammami et al. (2013)
2	Adjusted THI (THIadj) = [4.51 + THI − (1.992 × WS) + (0.0068 × RAD)] where THI = (0.8 × Tdb) + [RH × (Tdb −14.4)] + 46.4	
3	Heat load index (HLI) = 8.62 + (0.38 × RH) + (1.55 × BG) − (0.5 × WS) + e (2.4−WS), when BG > 25, HLI = 10.66 + (0.28 × RH) + (1.3 × BG) − WS, when BG ≤ 25, where BG = (1.33 × Tdb) − (2.65 × Tdb 0.5) is the predicted globe temperature (°C)	
4	TI4: Equivalent temperature index (ETI) = 27.88 − (0.45 × Tdb) + (0.010754 × Tdb 2) − (0.4905 × RH) + (0.00088 × RH2) + (1.1507 × WS) − (0.12644 × WS2) + [0.019876 × (Tdb × RH)]—[0.04631 × (Tdb × WS)]	
5	TI5: Environmental stress index (ESI) = (0.63 × Tdb) − (0.03 × RH) + (0.02 × RAD) + [0.0045 × (Tdb × RH)] − [0.073 × (0.1 + RAD) −1]	
6	TI6: comprehensive climate index (CCI) = RHadj + WSadj + RADadj, where RH e adj = × × db)	

^a^ WS, wind speed**;** RAD **=** solar radiation.

### 3.2 Physiological markers

Physiological markers such as respiration rates, rectal temperature, core-body temperature, skin temperature, sweating rate, panting score, drooling score, feed and water intake and production performance are significantly affected at different levels by heat stress (Wang et al., 2018; Pinto et al., 2020). These physiological parameters indicate the level of stress experienced by animals from climatic factors (Bouraoui et al., 2002; Kumar et al., 2017). Physiological responses to heat stress such as the increase in respiratory rate, panting score and heart rate are mechanisms mediated by the hypothalamus-pituitary-adrenal axis that animals use to cope with increases in environmental factors (Stumpf et al., 2021). These measures have also been proposed as heat tolerant traits (Carabaño et al., 2019). As such, genetic selection for regulation of physiological traits (e.g., rectal temperature) is one of crucial strategy to mitigate heat stress effects on dairy cow (Dikmen et al., 2012). However, genetics and heritability of those parameters is not well understood because of the difficulty to measure them in large number of animals and cost of routine accurate measurements of these parameters is high (Hammami et al., 2015). Smallholder farmers also regard these invasive measures with suspicion.

#### 3.2.1 Respiration rates and panting score

Respiratory rates (RR) and rectal temperature have long been used as heat stress markers (Pinto et al., 2020). Respiratory rate is a biomarker of heat stress in the hot environment. The normal RR is around 10–30 breaths/min in dairy cattle (Hady et al., 2018). Several studies, e.g., Pinto et al. (2020); Djelailia et al. (2021) reported that RR is usually measured by counting right thoracoabdominal movements for 15 or 30 s and multiplying the value obtained to get breaths per minute, bpm. Respiratory rates is measured by auscultation during 30 s and multiplying the obtained value by two to get the RR per minute (Bouraoui et al., 2002; Baena et al., 2018; Contreras-Jodar et al., 2019; Stumpf et al., 2021). In the study conducted by Osei-Amponsah et al. (2020) RR was measured in seconds taken for standing cows to make five flank movements. In livestock, RR increases owing to the activation of thermo-receptors in the animal’s skin when they are exposed to extreme air temperature. Such activation of the receptors, in turn, sends neural signals to the hypothalamus that raises respiratory rates to increase heat loss from the body by respiratory evaporations (Kumar et al., 2017).

Another indicator related to respiration rate is panting score (PS). The increase in PS causes an increase in the blood flow to the skin surface and thus facilitates heat loss (Kumar et al., 2017). In dairy cattle panting score (PS) ranges between 0 and 4.5 (Mader et al., 2006; Osei-Amponsah et al., 2020). It is assigned based on the panting level, presence or absence of drool, open mouth, and extended tongue (Osei-Amponsah et al., 2020). Panting score of a lactating dairy cattle is observed as drooling (saliva coming out of the cow’s mouth when she is not ruminating), open mouth (space between the lips visible when the cow is not ruminating), closed mouth (space between the lips is not visible), protruding tongue (the tip of the tongue crossed the edge of the bottom lip) and tongue inside the mouth (tongue rested on the floor of the mouth) (Tresoldi et al., 2016). Panting score is measured by visual observation and in a 0 to 4 scale (Stumpf et al., 2021). Panting is a more accessible tool and easier to measure than respiration rate, during identification of cows that experience high heat stress (Tresoldi et al., 2016). Dalcin et al. (2016) studied the effects of heat stress on PS for Holstein-Friesian dairy cattle in Brazil and found that animals with 75% Holstein-Friesian blood indicated 1.12 while those with 50% Holstein-Friesian blood had only 0.38 of pulse rate indicating that 50% Holstein-Friesian are more tolerant to the effects of heat stress than 75% Holstein-Friesian.

#### 3.2.2 Core-body temperature

When temperature and humidity values go above the level where evaporative cooling is effective, an increase in core body temperature (CBT) and hyperthermia occur (Cartwright et al., 2022). To detect the CBT of animals, the use of thermometers employs thermistors. Traditionally, CBT could be measured with a rectal thermometer, but this requires that animals have to be restrained, thus causing stress response arising from stress-induced hyperthermia that eventually reduces the precision of CBT determination (Wang et al., 2021). The accurate method for CBT measurement in dairy cattle is the use of infrared (IR) for animal sensors. The IR technology measures animal CBT remotely. Infrared thermography (IRT) is a simple, effective, on-site, and non-invasive approach that assess skin heat, emitted as infrared radiation and gives pictorial images without causing radiation exposure (Osei-Amponsah et al., 2020). The use of IRT to assess CBT is less laborious and time-consuming. Additionally, this approach is less-invasive and has higher automation potential than CBT assessment (Yan et al., 2021). The essence of IRT is to capture the infrared radiation released from the body if the temperature is greater than absolute zero. This implies at most, the CBT is noted (Wang et al., 2021). An infrared sensor exhibits the CBT owing to the whole radiation heat, but outermost variables can influence actual temperature, thus, rectifications are needed (Wang et al., 2021).

Currently, operating system in infrared tools give adjustable variables to reduce the effect from outermost variables. Variables that influence the IRT to measure heat radiation might be divided in three types: 1) Reflection, an usual issue in electronic tools, this might be remunerated if the correct reflection figure is provided; 2) Emissivity, the capacity of various instruments to discharge infrared energy, this can be broadly contrasting and must be adjusted; and 3) Environment, together with humidity and temperature, can also be regarded to regulate the device (Jeelani et al., 2019; Wang et al., 2021). Osei-Amponsah et al. (2020) used IRT to measure CBT for dairy cattle reared in Victoria, Melbourne, Australia. The IRT was used to assess surface temperature of dairy cattle and the heat stress imager used operated in the 8 to 14-μm spectral range and was calibrated to assess temperature from −30°C to 100°C (Jeelani et al., 2019). Baena et al. (2018) measured the CBT of the Angus cattle using a digital infrared thermometer in °C by targeting 20 cm below the vertebral column of the cattle. Garner et al. (2016) reported that CBT is lower in heat tolerant (HT) dairy cattle than heat-stressed (HS) animals.

#### 3.2.3 Rectal temperature

The rectal temperature (RT) is considered to be the best physiological trait to detect animal welfare in hot climate compared to RR (Kumar et al., 2017; Stumpf et al., 2021). The RT is measured using a veterinary digital thermometer and measurements are obtained directly from the rectal wall (Pinto et al., 2020). The accurate method to investigate heat stress is to determine RT above 39°C and RR lower than 60 beats/min (Wang et al., 2021). The veterinary digital thermometer is inserted at 60 mm into the rectum wall for 1 min and the temperatures are recorded to one decimal point (Djelailia et al., 2021). However, this approach for RT detection may not be suitable for long-term measurement (Wang et al., 2021). Rectal temperature can be measured using digital clinical thermometer (Contreras-Jodar et al., 2019) inserted 30 cm against the rectum wall for 3 minutes (Stumpf et al., 2021). In the study by Pinto et al. (2020), the RT of dairy cattle increased with a THI >70 starting at 38.4°C. In Tunisia, Bouraoui et al. (2002) measured RT by inserting a veterinary thermometer into the rectum of dairy cattle at approximately 60 mm for 1 min and the temperatures were recorded with one decimal point. In their study, RT was observed to increase when THI value increased from 68 to 78 as result of a daily increase of 0.5°C. In the study by Garner et al. (2016) in Australia, they recorded an increase in RT and vaginal temperature in their study, this remained consistent with average heat stress.

#### 3.2.4 Heart rates

Heart rates (HR) are commonly measured by putting a stethoscope between the fourth and sixth intercostal spaces in the breastbone region for 15 s and multiplying the obtained value by four to get beats per min, bpm (Pinto et al., 2020). Heart rates also are measured through auscultation during 30 s and multiplying the obtained value by two (Stumpf et al., 2021). It is sometimes measured using stethoscope for 1 min (Djelailia et al., 2021). In the study by Pinto et al. (2020), heart rates increased linearly from 81 bpm at a heat load threshold of 69 THI. Bouraoui et al. (2002) measured heart rates of dairy cattle in Tunisia using a stethoscope for 1 minute. In their study heart rates and RR increased by six beats and five inspirations per min, respectively. Such response changes are adaptive mechanisms initiated by the cow in an attempt to restore its thermal balance (Bouraoui et al., 2002). Heart rates was measured in beats per min using a stethoscope and a stopwatch for 30 s and multiplying the data by two to get the value in minutes (Dalcin et al., 2016). In their study, HR ranged from 81.74 breaths per minute in Holsteins, 77.47 breaths per minutes for 75% Holstein-Friesian and 52.86 breaths per minute for 50% Holstein-Friesian blood level (Dalcin et al., 2016). This indicates that 50% Holstein-Friesians are less affected by heat stress effects.

#### 3.2.5 Pulse rates

Pulse rate of the livestock species is measured by watching the pulsation of middle coccygeal artery at the base of the tail and the obtained data are recorded as pulse rate per 60 s (Berian, 2019). Gaafar et al. (2021) measured the pulse rate using a stethoscope. It was counted at the left thoracic region of the aortic arch, and obtained results were recorded as beats per 60 s. In Germany, Al-Kanaan et al. (2013) observed an increase in pulse rate with increasing of THI. In India, Pandey et al. (2017) studied the heat stress effects on pulse rate (beaths/min) of Tharparkar and Karan Fries at 40°C and 42°C and found a significant increase in the trait, whereby Tharparkar had 74.16 ± 0.87 while Karan Fries had 77.16 ± 0.60 beats/min at 40°C. This shows that heat stress has a significant effect on the pulse rates of both breeds in India, thereby decreasing their production performance. This also indicates low heat tolerance for Karan Fries compared to Tharparkar.

### 3.3 Other indicators of heat tolerance

#### 3.3.1 Milk yield and composition

Milk production decline is a good indicator of heat tolerance since it is most readily available as it can be acquired directly from an automatic milking system (Li et al., 2020). Milk yield is recorded immediately after hand milking or automatic milking. The fat, protein, lactose, solids-not-fats and other milk composition are determined using a milk composition analyzer, typically Lactosan (Zheng et al., 2021). Alternatively, milk fat, protein, lactose, solids-not-fats, total solids, and SCC can be determined by infrared spectroscopy analysis (Honig et al., 2012). Milk yield and composition measurements are less invasive and can be determined in large number of dairy cattle. Data recorded from these methods can be utilized in genomic selection of heat tolerant dairy cattle as countries without developed recording systems might prefer genomic evaluations using those data.

#### 3.3.2 Milk and blood metabolites

Haematological and other biofluid (e.g., milk, urine, plasma, and serum) parameters have been used to identify heat stress effect on dairy cattle productivity (Berian, 2019). Metabolomics, related to genomics, transcriptomics and proteomics, is considered as the method for detailed understanding of an individual’s metabolism (Fan et al., 2018). The strategy here is to determine which metabolites are produced when animals are heat stressed. These metabolites can be used for assessing heat tolerance in animals and hence, selection of heat-adaptive animals (Osei-Amponsah et al., 2019).

There are a variety of metabolomic approaches used to analyze animal biofluids, the most common being high performance liquid chromatography-mass spectrometry (HPLC-MS), gas chromatography–mass spectrometry (GC-MS), and proton nuclear magnetic resonance (^1^H-NMR) (Kim et al., 2021). Mass spectrometry have high sensitivity and can be utilized to quantify thousands of metabolites, but requires a large number of samples and has no reproducibility and reliability. Contrary, nuclear magnetic resonance seems to be appropriate as it has greater reproducibility and reliability, but has minimum sensitivity. It identifies metabolites from analysis of data using databases and libraries (Kim et al., 2021; Zhu et al., 2021).

The potential diagnostic biomarkers of heat stress in dairy cattle are mainly metabolites derived from amino acid, lipid and carbohydrate metabolism or gastro-intestinal microbiota-metabolites (Tian et al., 2016). Fan et al. (2018) reported 33 important metabolites which are heat stress biomarkers in dairy cows using liquid chromatography-mass spectrometry (LC-MS). Fifteen of these biomarkers (glucose, pyruvate, lactate, among others) are used in pathways including amino acid, glycolysis, TCA or nucleotide metabolism. This shows a heat stress effect on energy and nucleotide metabolism in lactating dairy cattle. Tian et al. (2016) used LC-MS and ^1^H-NMR and found that 53 potential biomarkers were up- or down-regulated in the heat stressed animals compared to non-heat stressed individuals. Fan et al. (2019) indicated that combining physiological parameters like THI, RT, and RR with identified heat stress milk metabolites provides relevant and reliable information about the heat stress effects in dairy cattle, and this can help the selection of genotypes and animals that are thermotolerant to heat stress.

#### 3.3.3 Oxidative stress biomarkers

Oxidative stress (OS) is a multiplication of reactive oxygen species (ROS) greater than the capacity of the body antioxidant physiological capacities to make safe neutralization (Hady et al., 2018; Gaafar et al., 2021). Heat stress in an animal cause a rise of OS, which in turn results in an increase of ROS in several cells and tissues of heat-stressed animals. Reactive oxygen species induced cellular deterioration is among the pathways in charge of the decrease of a livestock production owing to heat stress (Maibam et al., 2017). ROS are generated as heat stress response in the cell and these ROS led to cellular damage which causes cell death. A study by Sigdel et al. (2019) has shown that the gene MAPK8IP1 has implications in reducing heat stress caused by the generation of ROS and cellular apoptosis. According to Sigdel et al. (2019), gene CDKN1B is also implicated in this metabolic pathways. It is an OS similar gene, upregulated due to heat stress and has implications in apoptosis while eliminating heat induced protein group, thus decreasing stress at cellular proteotoxic. The body has a defense ability to manage OS as enzymatic superoxide dismutase (SOD), glutathione peroxidase (GSH), and catalase antioxidants, which increase due to heat stress (Jeelani et al., 2019). Hady et al. (2018) studied the heat stress the effects on OS for lactating dairy cattle and buffaloes in Egypt and found a significant effect of THI thresholds on glutathione peroxidase, superoxide dismutase, and catalase activities characterization during the study moment for those animal species. The enzymatic antioxidants, particularly metalloenzymes are primordial defense mechanisms that counterbalance the oxidative death of the intracellular components caused by ROS (Hady et al., 2018).

## 4 Genes responsible for heat tolerance in dairy cattle

Identifying genes influencing thermotolerance of dairy cattle is necessary for designing process for genetic improvement towards better health and production performance for low heat tolerant genotypes in hot regions and future climate change mitigations. Thermotolerance is a quantitative biomarker affected by multiple quantitative trait loci (Sigdel et al., 2019), and genomics research has characterized genomic sites that are crucial for body temperature regulation in dairy cattle (Rolf, 2015). Although, there is a dearth of information of the difference between thermotolerance in different animals, it is expected that thousands of metabolic mechanisms such as cellular, morphological, behavioural and neuro-endocrine organisms are involved (Cheruiyot et al., 2021). The major pathways increasing heat stress response in animals include chaperones, cochaperones, phosphorylation and kinase activation. See details in Srikanth et al. (2017); Fang et al. (2021). Rolf (2015) provided a comprehensive review on the genes regulating heat tolerance in dairy cattle. Most of these genes belong to the heat shock protein family (HSPH1, TRAP1, HSF1, HSPA6) (Baena et al., 2018), cell signaling (e.g., FGF4, ATP1B2; HSP90AA1) (Silpa et al., 2021), genes from HSP70 family (HSPA1A, HSPA4), subfamily HSP110 (HSPH1), HSP40 (DNAJA1, DNAJB1, DNAJA2) family (Srikanth et al., 2017). Another important gene controlling thermotolerance in dairy cattle is slick hair gene (*slick hair*) which regulates hair length. It was initially detected in Senepol and Corora cattle and mapped to chromosome 20. Animals with *slick hair* gene exhibit minimum skin temperatures under extreme climate change effects (Dikmen et al., 2008). During heat stress conditions, gene expression changes in dairy cattle include 1) activation of heat shock transcription factor 1 (HSF1); 2) increased expression of heat shock proteins (HSP) and reduced expression and synthesis of other proteins. See more in Collier et al. (2008).

The best strategy to reduce heat stress challenges, is to identify cattle with greater genetic composition for adaptation to climatic conditions of the tropics using crucial genes that have significant association with thermotolerance in animals such as HSF1 and HSPA6 genes identified on cattle chromosomes 14 and 3, respectively (Baena et al., 2018). It has been shown that HSPs protect hyperthermia (Badri et al., 2018), circulatory shock (Jeelani et al., 2019), and cerebral ischemia during heat strokes (Srikanth et al., 2017) and can be used for determining the mechanisms of protection and physiological responses of the body during heat stress in dairy cow (Maibam et al., 2017). HSP40 controls the ATPase action of HSP70 through interaction with the J section of the HSP70 proteins. The polymorphisms in Hsp90AA1 have been associated with heat tolerance among heat stressed phenotypes and genotypes (Badri et al., 2018). Expression of HSP70 gene is a marker for heat tolerant gene characterization in cattle and has numerous applications in the development of heat tolerant animals during climate change conditions (Osei-Amponsah et al., 2019). HSP70 can improve the mechanisms of protection of antioxidant enzyme using its chaperones and support in homeostatic mechanism under stress conditions. Expressions of HSP genes and genes from HSP70 family in different patterns across various seasons can help to breed for better adapted cattle (Osei-Amponsah et al., 2019). Srikanth et al. (2017) reported that RNA-seq analysis identified 8,567 genes. Among them, 465 genes were greatly upregulated (≥2-fold) while 49 genes were highly downregulated (≤2-fold) during heat stress conditions.

Transcriptome analysis play important role for identification of candidate genes related to complex traits such as heat tolerant traits. Using RNA-Seq, Deng et al. (2020) identified 200 differentially expressed genes (DEGs) in which most of the genes (including IGFB2, OAS2, and MX2) have significant implications with thermotolerance. These genes can be used in the genetic selection programme aiming to improve thermotolerance and reduce losses in dairy cattle production. Sigdel et al. (2019) identified CRY2 gene which is involved in heat tolerance and the knock down of CRY2 was shown to increase sensitivity to heat stress. Sigdel et al. (2019) also detected one genomic region located on BTA14 that explains an additive genetic variance for milk production greater than 0.5% during heat stress events in the first 2 parities. Notwithstanding, this genomic site contains genes HSF1 and EEF1D which also have implication in cellular response to extreme temperature and humidity effects.

HSF1 is a candidate heat tolerance gene which works by increasing expression of nascent HSPs, decline in fatty acid metabolism, results in the activation of endocrine system as responses to heat stress and promotes the refolding of denatured proteins (Rong et al., 2019; Deng et al., 2020). Increased expression of HSF1 gene causes inhibition of cell apoptosis during heat stress, improving cell survival percentage so as to reduce heat stress effects (Rong et al., 2019). Jeelani et al. (2019) also studied the effects of THI on expression levels of heat stress response genes in crossbred dairy cattle reared in sub-tropical regions of India and found that at minimum THI (≤74), the assertion of Hsf4, negatively regulating Hsp70 genes, was very high, but this reduced at THI 74. In this study, there was no greater assertion of Hsp70 genes such as HspA1A and HspA6, among others.

## 5 Genetic models for estimating genetic parameters for heat tolerance

Modeling heat tolerance in dairy cattle requires the development of heat stress models which determine the heat stress levels in regards to climatic data (Ravagnolo and Misztal, 2000). Globally, there has been a growing interest of using test day (TD) records to estimate heat tolerance genetic parameters, as this reduces the cost of recording dairy cattle performances, particularly those related to heat stress. Various TD models for statistical modeling of heat tolerance traits in livestock industry are discussed in Swalve (2000) with primary focus on production data. Those statistical models used for genetic evaluation of test-day milk records include repeatability models, multiple trait models and random regression models (RRM) (Cho et al., 2016). Repeatability models assume the same genetic correlation among all test day records (Cho et al., 2016). Repeatability models also assume that the additive genetic variance is continuous and the genetic correlation is one gradually which can lead to lower genetic gain than expected when used during genetic evaluation of longitudinal data (Ogawa and Satoh, 2021). Multiple trait models use every test-day record as different parameter while RRM consider covariate model of repeated test-day records progressively (Cho et al., 2016). Among those models, RRM are more suitable as they analyze each test-day record with assumption that genetic and non-genetic variances change along days in milk (DIM), parity, and other performance traits as do genetic and non-genetic correlations (Cho et al., 2016). Additionally, RRM are more appropriate as they include effects of several environmental parameters that affect dairy cattle in different ways during the lactation (Zavadilová et al., 2005).

Furthermore, RRM are used for the genetic evaluation of parameters recorded for long period of time (longitudinal data) during the life-time of a dairy cow as this permits evaluating the modification of a parameter as time function such as age or DIM (Salimiyekta et al., 2021). Random regression models are attractive for two reasons. First, they have large flexibility to fit smoother patterns of decay and second, they allow the use of eigen decomposition of the additive genetic covariance matrix to find selection criteria. Such selection criteria are not correlated among themselves and could help in the improvement of tolerance to heat stress with no effect on production level (Carabaño et al., 2014). For test day models used for selection on production, performance, and heat tolerance traits, one of the eigen functions has been associated with the persistency of lactations. These eigen functions have been also advocated as a selection criterion for this trait to avoid problems of antagonistic relationship between production level and other persistency measures (Carabaño et al., 2014).

In RRM, animal has two genetic effects i.e., a regular effect that corresponds to animal performance in less stress environment and a heat-stress effect corresponding to the milk yield decline in heat-stress environment. Thus, the genetic and permanent environmental correlations among productions at several THI and DIM tend to be less than one (Ravagnolo and Misztal, 2000; Bohlouli et al., 2013). Table 3 provides examples of genetic models used to study heat tolerance in dairy cattle. Included among them are RRM, Broken Line (BL) model, Legendre polynomial (LP) and Reaction norm (RN) models. RRM are prominent during complex statistical modeling based on a limited number of observations (Brügemann et al., 2013). RRM permits studying the variation of genetic parameters components during the life-time of a particular dependent covariate like DIM. Legendre polynomial functions express the progress of milk production parameters over a complete lactation of dairy cow in several environmental situations (Hammami et al., 2008). A genetic evaluation of these indicators lead to production of similar genetic variance components that should be used in selective breeding programmes (Sánchez-Molano et al., 2020). Third order Legendre polynomial functions are adequate for estimating variance components because they accompany the form of the genetic and permanent environmental variances above performance parameter with high precision (Salimiyekta et al., 2021). Those models allow studying the additive genetic effects as a model of environmental situation by estimating genetic parameters above the scale of an environment-dependent covariate (Usala et al., 2021). RRM using polynomial functions are used to detect the THI thresholds at which production begins to decline and also the level at which production decreases after some unknown THI values (Sánchez-Molano et al., 2020; Mbuthia et al., 2021). In statistical models, tolerance to heat stress should be adapted in relation to a RN function where the trait is altered as a linear equation of an environmental factor (e.g., THI or humidity). Generally, the environmental factor effect is a dummy variable, put at zero in case THI < TH_0_ and to THI − TH_0_ in case THI > TH_0_ (Macciotta et al., 2017).

**TABLE 3:** Examples of genetic models used to study heat tolerance in dairy cattle.

Genetic model	Species	Country	References
Random regression models	Dual-purpose Guzerá cattle, Holstein Friesian, Jersey cattle	Brazil, Belgium, Luxembourg, Spain, Slovenia, Italy, United States, Thailand, Iran	(Hammami et al. (2013); Aguilar et al., (2009); Boonkum et al., (2011); Bohlouli et al., (2013); Hammami et al. (2013); Bernabucci et al., (2014); Hammami et al., (2015); Carabaño et al., (2016); Santana et al., (2017); Negri et al., (2021)
Broken Line and Legendre Polynomials functions	Holstein, Friesians cattle	Belgium, Luxembourg, Spain, Slovenia, China	Carabaño et al. (2016), Li et al. (2020)
Reaction norm models	Holstein, Jersey cattle	Kenya, Tanzania	Ekine-Dzivenu et al. (2020), Mbuthia et al. (2021)

Hammami et al. (2013) applied RRM for Holstein Friesian dairy cattle in Belgium to determine losses associated with milk production traits owing to heat stress and THI thresholds using a broken-line regression model. In this study, daily fat productions appeared to decline consistently as THI thresholds increased while somatic cell score productions were indicated by greater thresholds at both minimum and maximum THI scales, with high response to cold stress for obvious THI indicators. Carabaño et al. (2016) evaluated the THI effect on milk production and composition of highly selected dairy cattle reared in Walloon Region of Belgium, Luxembourg, and Southern Spain using traditional BL model correlated with quadratic and cubic functions which were ideal for following production response to escalating heat stress. A cubic polynomial function allowing for individual variation in pattern of response and THI_avg_ as heat stress measure indicated the best statistical features. Superior/inferior producing dairy cattle indicated reduced/increased constant production over the THI range. Under the broken line model, a value for the defective threshold or comfort point of 72 for the commonly used THI in cattle is extensively adopted (Carabaño et al., 2016). However, polynomial models give greater flexibility in responses than the BL models, to study milk yield changes to escalating heat stress (Carabaño et al., 2016). Genetic correlations between traits can change significantly amid of covariates applied for statistical computing when applying RRM (Brügemann et al., 2013). Brügemann et al. (2013) used RRM when regressing THI applying third-order-Legendre Polynomials for conception rate (CR) and somatic cell score to quantify the breaking points for CR and estimate genetic components for CR amid of THI. They found that at THI˃ 83, heat stress occurred and heritabilities started to increase. Further, significant decrease in CR for dairy cattle with shallow and normal degree of production were perceived in part of situations for THI greater than 65.

## 6 Genetic parameters for heat tolerance in dairy cattle

To advance a viable breeding and dairying scheme, it is crucial to estimate the genetic parameters and variance components for production, reproduction and heat tolerance traits (Chawala et al., 2017). Thus, estimated genetic correlations allying physiological biomarkers of heat stress and other important traits is important for the design of dairy cow breeding schemes seeking to improve thermotolerance (Luo et al., 2021). Table 4 provides examples of quantitative genetic models used to estimate genetic parameters for measuring heat tolerance in dairy cattle. Among those models include random regression models, reaction norm models and bivariate animal models (Table 4). Random regression models fitting reaction norm equations give details about the variation in individual production owing to climate change, with a horizontal reaction norm being indicator of a heat tolerant animal, whose production doesn’t change by weather (Sánchez-Molano et al., 2020). Headlines of sole animal reaction norms, like slopes at a specific value of the curve assist as resilience indicators. As such, genetic analysis of those phenotypes lead to production of approximate genetic parameters and breeding values that are applied for genetic selection for heat tolerance (Sánchez-Molano et al., 2020).

**TABLE 4:** Examples of genetic parameters used for measuring heat tolerance in dairy cattle.

Genetic models	Traits	Genetic parameters	Genetic values	Country	References
Random regression, reaction norm, Bivariate animal models	Milk production, fertility, calving age, calving season, udder health	Heritability, repeatability, breeding values, and genetic correlations	σ^2^_a_ = 0.08–0.22; r_g_ = −0.24 to −0.56, h^2^_a_ = 0.13–0.28, h^2^_thi_ = 0.09–0.37; r_g_˃80; R = 0.021–0.411; h^2^ = 0.13–073; h^2^ = 0.111–0.176	Belgium, Brazil, Iran, Italy, South Korea, Thailand	Boonkum et al. (2011), Bernabucci et al. (2014), Hammami et al. (2015), Rahbar et al. (2016), Santana et al. (2017), Lee et al. (2019)
Random regression models	Test day milk yield	Heritability, genetic and phenotypic correlations	h^2^ = 0.15–0.21	Brazil	Negri et al. (2021)

σ^2^_a_ **=** genetic correlations among parities for additive effects of heat stress (Thailand), h^2^ = heritability; r_g_ = genetic correlations, h^2^_a_ = general effect heritability, h^2^_thi_ = heat stress heritability (Italy); r_g_ = Estimates of genetic correlations within traits between cold and hot environment; R = Repeatability of fertility traits for first service to insemination outcome (Iran); h^2^ = Estimates of heritabilities within milk production traits at different THI, thresholds in first, second and third lactation stages (Brazil); h^2^ = heritability estimates of milk yield ranged from 0.111 t^0^ 0.176 (average 0.128). It decreased slightly as THI, increased, and began to increase at THI, of 79 (South Korea).

Estimation of genetic parameters for milk production traits has been done using principal component analysis (Macciotta et al., 2017). Their study found that heritability had a moderate to high level across all the traits. Rahbar et al. (2016) estimated the heritability, repeatability, the genetic and phenotypic correlation between fertility traits (success in first service (FS), gestation length (GL), number of inseminations (NI), insemination outcomes/IO, calving interval (CI), calving birth weight (CBW) and days in open (DO)) in Iran. Genetic and phenotypic correlations between those traits were estimated using bivariate animal linear models (Rahbar et al., 2016). In this study, the heritability (h^2^) and repeatability for GL and IO was less affected by heat stress than other traits, while DO and SF were highly affected by heat stress effects. Hammami et al. (2015) estimated the genetic parameters for production, udder health, and milk composition traits that are associated with tolerance for heat stress using linear RN models to estimate the intercept and slope patterns of 23 parameters to escalating THI thresholds. In this study, it was found that main production and fatty acids (FA) parameters showed phenotypic and genetic decreases as THI escalated, whereas other FA groups such as unsaturated FA and long-chain FA escalated with THI.

Bernabucci et al. (2014) estimated the genetic parameters of heat stress effects on milk yield traits for Italian dairy cattle and found that THI had a persistent effect on all production traits. Negri et al. (2021) estimated the heritability for the test day milk production regressed to the THI and found that the value of h^2^ ranged from 0.15 to 0.21 when using the Legendre polynomial equations. Nine analyses using two-trait RRM were performed to approximate variance components and genetic parameters for milk production traits (milk yield×fat percentage (MYxF%), milk yield×protein percentage (MYxP%) and milk yield×somatic cell score (SCS) in the study conducted in Brazil (Santana et al., 2017). Generally, the heritability estimates were superior for minimum THI thresholds and prolonged DIM. On the other hand, the heritability estimates for SCS increased with escalating THI thresholds in the second and third lactations. For milk yield in all lactations, the genetic correlations allying higher THI thresholds were elevated, up to 0.90, 0.76 and 0.68, in first, second and third lactation, respectively (Santana et al., 2017). For fats and protein percentages, the genetic correlations were at all times 0.80 or superior in the three lactations. The genetic correlations among milk production traits changed over the THI range and lactations (Santana et al., 2017).

## 7 Genetic relationships between indicators of heat tolerance and performance traits

Identification of reliable correlations between environmental variables and animals’ response to heat tolerance is an initial point for the statistical simulation of their effects on the subsequent welfare and milk production of a dairy cattle (Mylostyvyi and Chernenko, 2019). Genetic correlations allying milk yield parameters estimated breeding values (EBVs) (e.g., milk) with reproductive traits was estimated from dairy cattle reared in Walloon Region of Belgium (Mineur et al., 2018). It was indicated that an inclusion of novel milk production parameters with traditional performance parameters, genetic relationships underlying EBVs and also GEBVs for those milk production parameters and reproductive parameters improved significantly. According to Rahbar et al. (2016), the reproductive traits show low heritability (h^2^ < 10%) most often in Holstein dairy cows, indicating that fertility is overwhelmed mainly by THI. In the study by Hammami et al. (2015), estimates of genetic correlations of the same traits collected in cold and hot environments of Walloon Region in Belgium, indicated that the correlations were above 0.5. On the other hand, lower estimates were obtained for SCS and fat contents, implying that dairy cattle with greatest genetic value for these parameters in cold regions don’t have the greatest value for the same parameters in hot regions. Similarly; Mylostyvyi and Chernenko (2019), estimated the correlations between environmental parameters and the productive traits of dairy cattle and obtained a positive correlation between environmental conditions and days in milk yields as well as between relative humidity (RH) and days in milk and between RH and milk fats. Aguilar et al. (2009) estimated genetic parameters for milk production and productive parameters during extreme climatic conditions and found adverse genetic correlations between THI and those production performance parameters. Bernabucci et al. (2014) found antagonistic genetic relationship estimates between production and heat stress across parities and parameters, indicating that genetic selection for increased milk production traits alone can cause a decrease in the genetic value for thermotolerance, except that production indicator itself is utilized as a criteria for selection in a mass index. Bouraoui et al. (2002) conducted a 2 year research work, and found unfavourable relationships between milk production and daily THI, with a decline of 0.41 Kg per cow per day for every THI unit rise beyond 69. Boonkum et al. (2011) estimated heat additive variance for third parity *versus* first parity on Thai Holstein crossbreds and found a greater genetic variability related to heat stress. In their study genetic correlations among parities were ≥0.88 for test-day milk yield without consideration of heat stress, but were ≤0.22 when heat stress was considered. In Brazil, Santana et al. (2017) estimated the genetic relationships between milk production and SCS and found that the genetic correlation estimates revealed apparent shift of decline with escalating THI merits.

## 8 Conclusion

This review provides an insight on the genes and models for estimating genetic parameters for heat tolerance in dairy cattle. It indicated various biomarkers used to measure heat tolerance in dairy cattle. Among them, milk yield decline, alteration in milk composition (fats, proteins, lactose, solids-not-fats), physiological parameters (core body temperature, rectal temperature, respiration rate, panting score, drooling score, and heart rate), oxidative biomarkers and genes from heat shock protein family were identified as potential biomarkers used for measuring heat tolerance in dairy cattle. This review indicated that continued genetic selection for milk production traits without involving heat tolerance traits in the selection objective leads to high susceptibility to heat stress. The crucial genes responsible for heat tolerance belong to heat shock protein family, cell signaling and are involved in chaperones, cochaperones, immune response and apoptosis pathways. *Slick hair* gene is also an important gene controlling heat tolerance in dairy cattle and should be introduced in dairy cattle breeds through crossbreeding as an effort to improve heat tolerance. This review highlighted various approaches to estimate the genetic parameters for heat tolerance in dairy cattle. The RRM fitting Legendre polynomial functions and reaction norm models are appropriate models used to estimate genetic parameters for heat tolerance in dairy cattle. It was revealed that there are unfavourable genetic relationship estimates between production parameters and heat tolerance across parities and traits. This indicates that genetic selection aiming for increased milk production parameters alone can cause a decline in the genetic values for thermotolerance. This review contributes to a better understanding of the genes, quantitative genetic models and novel phenotypes underlying milk yield traits under climate change effects. This provides opportunities to breed heat tolerant dairy cattle breeds while minimizing costs for cooling technologies and income losses. In future, there is a need to develop selection strategies for identifying thermotolerant animals and estimating heat tolerance breeding values. Furthermore, THI thresholds should be included in the selection index as a criterion for selection, particularly for dairy cattle reared in hot regions in which effectiveness can continue to degenerate for long time.
